# Transcriptome datasets of neural progenitors and neurons differentiated from induced pluripotent stem cells of healthy donors and Parkinson's disease patients with mutations in the *PARK2* gene

**DOI:** 10.1016/j.dib.2022.107958

**Published:** 2022-02-16

**Authors:** Ekaterina Novosadova, Ksenia Anufrieva, Elizaveta Kazantseva, Elena Arsenyeva, Viya Fedoseyeva, Ekaterina Stepanenko, Daniil Poberezhniy, Sergey Illarioshkin, Lyudmila Novosadova, Tatiana Gerasimova, Valentina Nenasheva, Igor Grivennikov, Maria Lagarkova, Vyacheslav Tarantul

**Affiliations:** a Institute of Molecular Genetics of National Research Centre “Kurchatov Institute”, Moscow, Russia; b Federal Research and Clinical Center of Physical Chemical Medicine of the Federal Medical and Biological Agency of the Russian Federation, Moscow, Russia; c Faculty of Biotechnology and Industrial Ecology, D.I. Mendeleyev University of Chemical Technology of Russia, Moscow, Russia; d Research Center of Neurology, Moscow, Russia

**Keywords:** Induced pluripotent stem cells, Neural progenitors, Terminally differentiated neurons, Parkinson's disease, NextSeq 500 System (Illumina), *PARK2*, Transcriptome analysis

## Abstract

Parkinson's disease (PD) is a complex systemic disorder caused by neurodegenerative processes in the brain that are mainly characterized by progressive loss of dopaminergic neurons in the substantia nigra. About 10% of PD cases have been linked to specific gene mutations (Zafar and Yaddanapudi, 2022) including the *PARK2* gene that encodes a RING domain-containing E3 ubiquitin ligase Parkin. PD-Parkin patients have a younger onset, longer disease duration, and more severe clinical symptoms in comparison to PD patients with unknown causative PD mutations (Zhou et al., 2020). Induced pluripotent stem cells (iPSCs) are considered to be a powerful tool for disease modeling. To evaluate how mutations in *PARK2* contribute to PD development, iPSC lines were obtained from three healthy donors and three PD patients with different mutations in the *PARK2* gene. iPSC lines were differentiated consequently into neural progenitors (NPs) and then into terminally differentiated neurons (DNs). The data presented in this article were generated on an NextSeq 500 System (Illumina) and include transcriptome profiles for NPs and DNs of healthy donors and PD patients with mutations in the *PARK2* gene. Top10 up- and down-regulated differentially expressed genes in NPs and DNs of patients with PD compared to healthy donors were also presented. A comparative transcriptome analysis of neuronal derivatives of healthy donors and PD patients allows to examine the contributions of the *PARK2* gene mutations to PD pathogenesis.

## Specifications Table

**Table:** 

Subject	Cell biology
Specific subject area	Neural progenitors (NPs) and terminally differentiated neurons (DNs) generated from induced pluripotent stem cells (iPSC) of healthy donors and PD patients with different *PARK2* mutations
Type of data	Transcriptomic data Tables Figures
How data were acquired	Transcriptome data were obtained using the NextSeq 500 System (Illumina)
Data format	Raw RNA sequencing data in FASTQ format
Parameters for data collection	NPs and DNs differentiated from iPSC lines of three healthy donors and three PD patients with different mutations in the *PARK2* gene were harvested for RNA-Seq based transcriptomic analysis.
Description of data collection	Total RNA from each sample in triplicate was extracted using the RNeasy Micro Kit (Qiagen, USA). RNA quality was checked using the 2100 Bioanalyzer (Agilent, USA). Enrichment of polyadenylated RNA and library preparation was performed with NEB Next Ultra II Directional RNA Library Prep (NEB, USA) according to the manufacturer's protocol. Samples were sequenced on the NextSeq 500 System (Illumina, USA) with the NextSeq 500/550 High Output Kit v2.5 (75 Cycles).
Data source location	Institute of Molecular Genetics of National Research Centre “Kurchatov Institute” Moscow Russia
Data accessibility	Repository name: Gene Expression Omnibus (GEO) Data identification number: GSE181029 Direct URL to data: https://www.ncbi.nlm.nih.gov/geo/query/acc.cgi?acc=GSE181029

## Value of the Data

• The transcriptomic datasets generated are useful for identifying genes involved in the PD pathogenesis and determining mechanisms of PD onset associated with mutations in *PARK2. PARK2* was selected for analysis as mutations in this gene have been shown to cause autosomal recessive early onset PD [2], [3].
• These data are valuable for researchers who investigate gene network in the process of neuronal differentiation and molecular mechanisms involved in PD development. Analysis of healthy NPs and DNs vs. PD NPs and DNs can be important for research in disease modeling, autologous iPSCs implantation [4] and genetic methods of disease correction.
• These data may be used to perform multilevel comparative transcriptomic analysis of NPs and neurons in healthy donors and PD patients with mutations in various *PARK2* exons, as well as to evaluate transcriptional features that healthy or PD NPs acquire during differentiation into mature neurons.

## 1 Data Description

**Fig. 1:**
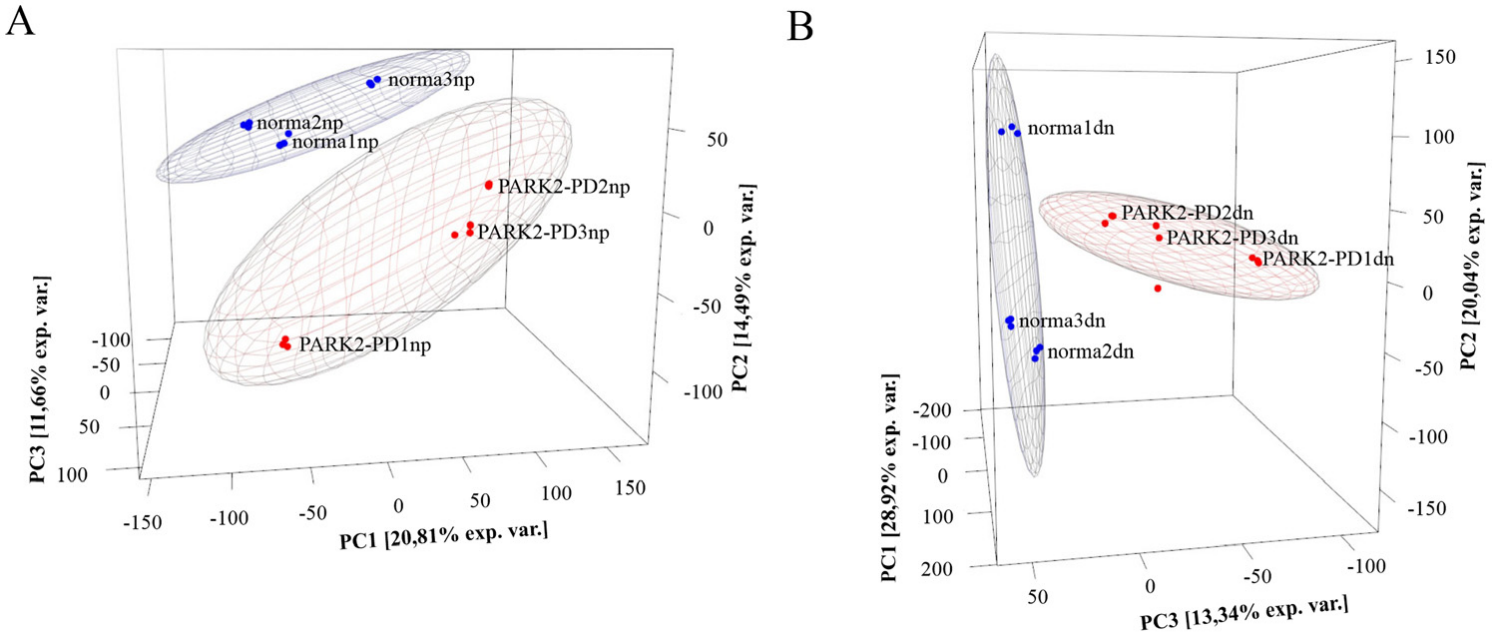
Principal component analysis of normalized data by DESeq2 package. (A), PCA for NP transcriptome profiles. (B), PCA for DN transcriptome profiles. The blue dots represent three healthy donors, and the red dots represent three PD patients (each in triplicate).

PD affects at least 1% of the world population over 60. About 10% of PD cases have been linked to specific gene mutations, mainly in young people [1]. Research on contribution of gene mutations to PD pathogenesis is thus highly significant. One of PD-associated genes, *PARK2*, is located in a region susceptible to form gaps, breaks, and rearrangements [5], so the different mutations in this gene are in the focus of interest. To date, there are few reports on the transcriptome of neural derivatives differentiated from PD patients’ iPSCs. We obtained the iPSC-derived neural cells at different differentiation stages: neural progenitors (NPs) and terminally differentiated neurons (DNs) from both healthy donors and PD patients with the *PARK2* gene mutations. According to publications, *PARK2* mutations most commonly occur in exons 3–6 [6]. Our data covers PD patients with deletions of the 2nd and 8th exons. iPSCs from three healthy donors and three PD patients with different mutations in *PARK2* were differentiated into uncommitted NPs, and then into mature DNs (Table 1, Supplementary Figs. S1–S4). Whole transcriptome profiles of these cell populations were generated using NextSeq 500 System (Illumina, USA). The datasets contain raw sequence data converted into the FASTQ format. Raw transcriptome sequence reads were deposited into the NCBI GEO database (Accession number GSE181029). Reads were trimmed for quality (Supplementary Table S1); paired reads were trimmed using Trimmomatic (v. 0.35) [7] the first 1 and last 1 bases. Trimmed RNA-seq reads were quantified against Homo Sapiens GRCh38.13 genome annotation at the transcript level using Salmon (v.1.4) [8]. Results were aggregated to gene level using the R package tximport [9]. R packages FactoMineR [10] and rgl (0.108.3) [11] were used for PCA analysis and data visualization, respectively. Fig. 1. (A and B) visualizes the principal component analysis (PCA) of NP and DN transcriptome profiles, respectively. Fig. 1A demonstrates that “healthy” NPs (blue dots) form a separate compact cluster. Differentially expressed genes were identified using the R package DESeq2 [12] Table 2. presents Top10 differentially expressed genes in NPs of PD patients as compared to HD. Fig. 1B shows that DNs from HD and PD patients form two different clusters. Table 3 presents Top10 differentially expressed genes in DNs of PD patients as compared to HD.

**Table 1:** Description of cell lines used.

Designation	Description of PD patientsand healthy donors	Genotype	NPcell line name	DNcell line name
norma1 (IPSRG2L) [13]	Healthy male, 60 years	normal	NP RG2L	DN RG2L

norma2 (IPSHD1.1S) [13]	Healthy female, 18 years	normal	NP HD 1.1S	DN HD 1.1S

norma3 (IPSFD3.9L) [13]	Healthy female, 26 years	normal	NP FD 3.9 L	DN FD 3.9L

PARK2-PD1 (IPSPDP1.5L) [13]	Male with PD, the beginning of disease—23 years, biopsy—64 years	(del 202-203 AG; IVS1+1G/A)*PARK2*	NP PDL 1.5L	DN PDL 1.5L

PARK2-PD2 (IPSPDPS8)	Female with PD, the beginning of disease—30 years, biopsy—41 years	EX8 del *PARK2*	NP PDS13	DN PDS13

PARK2-PD3 (IPSPDPS2d)	Male with PD, the beginning of disease—38 years, biopsy—40 years	het EX2 del *PARK2*	NP PDS14	DN PDS14

**Table 2:** Top10 up- and down-regulated differentially expressed genes in PD NPs compared to HD NPs.

Ensembl_ID	Gene symbol	log_2_FC (gene expression in PD NPs/gene expression in HD NPs)	*p-*value with FDR adjustment
ENSG00000165970.11	*SLC6A5*	23,1	7.0 × 10^−13^
ENSG00000185610.6	*DBX2*	23.0	4.9 × 10^−16^
ENSG00000171564.11	*FGB*	22.7	2.1 × 10^−12^
ENSG00000115263.14	*GCG*	22.6	2.8 × 10^−12^
ENSG00000107807.12	*TLX1*	22.5	3.1 × 10^−12^
ENSG00000165556.9	*CDX2*	22.4	4.6 × 10^−12^
ENSG00000075388.3	*FGF4*	22.0	1.3 × 10^−11^
ENSG00000170689.9	*HOXB9*	13.3	4.6 × 10^−09^
ENSG00000120068.6	*HOXB8*	12.0	5.7 × 10^−09^
ENSG00000143839.14	*REN*	11.9	1.4 × 10^−04^
ENSG00000163762.6	*TM4SF18*	−9.5	1.63 × 10^−73^
ENSG00000153266.12	*FEZF2*	−7.8	5.23 × 10^−15^
ENSG00000142700.11	*DMRTA2*	−7.7	3.27 × 10^−29^
ENSG00000231609.6	*AC007098,1*	−7.2	1.57 × 10^−15^
ENSG00000257501.6	*AC007424,1*	−7.1	5.43 × 10^−45^
ENSG00000110077.14	*MS4A6A*	−7.0	2.08 × 10^−62^
ENSG00000075290.7	*WNT8B*	−7.0	3.20 × 10^−30^
ENSG00000115507.9	*OTX1*	−6.9	6.69 × 10^−28^
ENSG00000254300.1	*LINC01111*	−6.6	5.19 × 10^−10^
ENSG00000161281.10	*COX7A1*	−6.5	1.47 × 10^−12^

**Table 3:** Top10 up- and down-regulated differentially expressed genes in PD DNs compared to HD DNs.

Ensembl_ID	Gene symbol	log_2_FC (gene expression in PD DNs/gene expression in HD DNs)	*p-*value with FDR adjustment
ENSG00000170689.9	*HOXB9*	10,7	5.9 × 10^−15^
ENSG00000122592.7	*HOXA7*	9.4	1.7 × 10^−12^
ENSG00000165092.12	*ALDH1A1*	7.2	1.3 × 10^−09^
ENSG00000133110.14	*POSTN*	6.8	4.3 × 10^−22^
ENSG00000163145.12	*C1QTNF7*	6.7	1.4 × 10^−05^
ENSG00000173641.17	*HSPB7*	6.5	3.9 × 10^−08^
ENSG00000132854.18	*KANK4*	6.5	2.6 × 10^−12^
ENSG00000129009.12	*ISLR*	6.4	4.4 × 10^−12^
ENSG00000184058.14	*TBX1*	6.4	6.6 × 10^−06^
ENSG00000112837.16	*TBX18*	5.8	1.3 × 10^−08^
ENSG00000131095.12	*GFAP*	−8.2	4.8 × 10^−12^
ENSG00000196126.11	*HLA-DRB1*	−7.2	5.8 × 10^−08^
ENSG00000174059.16	*CD34*	−6.2	2.4 × 10^−08^
ENSG00000171885.14	*AQP4*	−6.0	7.8 × 10^−10^
ENSG00000047457.13	*CP*	−5.8	4.0 × 10^−12^
ENSG00000243649.8	*CFB*	−5.6	1.7 × 10^−07^
ENSG00000147485.12	*PXDNL*	−5.5	2.4 × 10^−09^
ENSG00000118004.17	*COLEC11*	−5.4	3.3 × 10^−07^
ENSG00000165949.12	*IFI27*	−5.3	1.4 × 10^−08^
ENSG00000093010.13	*COMT*	−4.6	5.8 × 10^−40^

## 2 Experimental Design, Materials and Methods

### 2.1 Ethics statement

The study complies with the Declaration of Helsinki and was performed following approval by the Ethic Committee of the Research Center of Neurology. Written informed consent was obtained from every patient and healthy donor.

### 2.2 Derivation of IPSPDPS8 and IPSPDPS2d cell lines

iPSCs were derived from human skin fibroblasts of patients carrying mutations in the *PARK2* gene using CytoTune™-iPS 2.0 Sendai Reprogramming Kit (Thermo Fisher, USA). The mutations were localized using MLPA method with subsequent sequencing. The obtained iPSCs expressed the necessary pattern of specific pluripotency-associated genes: SSEA-4, Oct-4 (Supplementary Fig. S1) and possessed a normal karyotype. The iPSCs could produce the derivatives of three embryonic germ layers. Spontaneously differentiated iPSCs were stained with antibodies for the markers of the derivatives of three germ layers (ectoderm-TUBB3, mesoderm-Desmin, entoderm–AFP) (Supplementary Fig. S2). iPSC lines were cultured in XF Medium (Sartorius, Germany) on Matrigel-coated substrates (Corning, USA). Cells were investigated with an AxioImager Z1 fluorescence microscope equipped with an AxioCam HRM camera using AxioVision 4.8 software (Zeiss, Germany). Immunofluorescence staining was performed according to a previously described method [13].

### 2.3 Differentiation of iPSCs into NPs

iPSC cultures generated from somatic cells of healthy and PD donors [13,14] were maintained in hPSC XF Medium (Sartorius, Germany). To start iPSC differentiation in neuronal direction [15] the culture medium was replaced with medium for NPs (DMEM/F12 medium supplemented with 2% serum replacement (Gibco, USA) and a mix of factors: 1 mM non-essential amino acids (Paneco, Russian Federation), 2 mM L-glutamine (ICN Biomedicals Inc, USA), penicillin-streptomycin(50 U/ml; 50 µg/ml) (Paneco, Russian Federation), 1% N2 supplement (Life Technologies, USA), 10 µM SB431542 (Stemgent, USA), and 80 ng recombinant Noggin (Peprotech, USA)). After 10–14 days of culturing, neural rosettes with specific “ridges” were formed. Rosettes were mechanically transferred to a 24-well plate with extremely low adhesion (Corning Life Sciences, USA) for 3–5 days until neurospheres were formed. Neurospheres were collected and treated with 0.05% trypsin. After trypsin inactivation in DMEM with 10% FBS, cells were resuspended in growth medium for NPs, and transferred to Petri dishes coated with Matrigel (Corning, USA). Prior to the first passage, cell cultures were morphologically characterized and stained with Sox1 antibodies (Abcam, USA) since Sox1 protein is a specific marker of uncommitted NPs [15] (Supplementary Fig. S3).

### 2.4 Differentiation of NPs into DNs

For differentiation into terminally differentiated neurons (DNs), mainly dopaminergic, the NPs were cultivated in DMEM/F12 medium containing 2% serum replacement (Gibco, USA), 1 mM non-essential amino acids (Paneco, Russian Federation), 2 mM L-glutamine (ICN Biomedicals Inc, USA), penicillin-streptomycin (50 U/ml; 50 µg/ml) (Paneco, Russian Federation), 1% В27 supplement (Life Technologies, USA), 100 ng/ml recombinant Shh, 20 ng/ml recombinant FGF8, purmorphamine 2 µM all from (Peprotech, USA). After 10 days, the medium was changed to DMEM/F12, containing 2% serum replacement (Gibco, USA), 1 mM non-essential amino acids (Paneco, Russian Federation), 2 mM L-glutamine (ICN Biomedicals Inc, USA), penicillin-streptomycin (50 U/ml; 50 µg/ml) (Paneco, Russian Federation), 1% В27 supplement (Life Technologies, USA), 5 µM forskolin (Stemgent, USA), 20 ng/ml BDNF, 20 ng/ml GDNF, 200 µM ascorbic acid all from (Peprotech, USA) for the following 24 days. Expression of dopaminergic neuron markers (beta-III-tubulin and tyrosine hydroxylase) in cell culture obtained was confirmed by staining the cells with specific antibodies (Abcam, USA) (Supplementary Fig. S4), and specific mRNA expression was assessed using qRT-PCR [15].

### 2.5 Transcriptome data profiling

Total RNA from NP and DN cultures in triplicate was extracted using the RNeasy Micro Kit (Qiagen, USA) followed by treatment with DNAse I (Qiagen, USA). The reaction was purified with the PureLink RNA Mini Kit (ThermoFisher, USA). RNA samples quality was checked using 2100 bioanalyzer (Agilent, USA). Enrichment of polyadenylated RNA and library preparation was performed with NEB Next Ultra II Directional RNA Library Prep (NEB, USA) according to the manufacturer's protocol. Samples were sequenced on the NextSeq 500 System (Illumina, USA) with the NextSeq 500/550 High Output Kit v2.5 (75 Cycles).

### 2.6 RNAseq data analysis

Raw sequence data were converted to the FASTQ format using the bcl2fastq software (Illumina). Reads were trimmed for quality (Supplementary Table S1); paired reads were trimmed using Trimmomatic (v. 0.35) [7] the first 1 and last 1 bases. Trimmed RNAseq reads were quantified against Homo Sapiens GRCh38.13 genome annotation at the transcript level using Salmon (v.1.4) [8]. Results were aggregated to gene level using the R package tximport [9]. Differentially expressed genes were identified using the R package DESeq2 [12]. R packages FactoMineR [10] and rgl (0.108.3) [11] were used for PCA analysis and data visualization, respectively. All software packages and libraries used can be accessed via the GitHub repository: https://github.com/ksenia1602/scripts_for_articles/tree/main/scripts_Data_in_Brief_artcicle_NP_ND_from_IPS.

## CRediT authorship contribution statement

**Ekaterina Novosadova:** Conceptualization, Investigation, Resources, Visualization. **Ksenia Anufrieva:** Methodology, Software, Validation, Formal analysis, Investigation, Resources. **Elizaveta Kazantseva:** Investigation. **Elena Arsenyeva:** Investigation. **Viya Fedoseyeva:** Formal analysis, Investigation. **Ekaterina Stepanenko:** Investigation. **Daniil Poberezhniy:** Formal analysis, Investigation, Methodology, Software. **Sergey Illarioshkin:** Resources. **Lyudmila Novosadova:** Investigation. **Tatiana Gerasimova:** Investigation, Visualization, Writing – review & editing. **Valentina Nenasheva:** Data curation, Writing – original draft, Writing – review & editing, Funding acquisition. **Igor Grivennikov:** Writing – review & editing. **Maria Lagarkova:** Data curation, Project administration. **Vyacheslav Tarantul:** Data curation, Writing – review & editing, Project administration, Funding acquisition.

## Declaration of Competing Interest

The authors declare that they have no known competing financial interests or personal relationships which have or could be perceived to have influenced the work reported in this article.

## Appendix Supplementary Materials

Image, application 1
