# Supplementary material for: Transcriptome datasets of neural progenitors and neurons differentiated from induced pluripotent stem cells of healthy donors and Parkinson's disease patients with mutations in the PARK2 gene
Source: Data Brief. 2022 Feb 16;41:107958. doi: 10.1016/j.dib.2022.107958 (PMC8867054; doi:10.1016/j.dib.2022.107958)
Supplement: Supplementary file 1 [file mmc1.pptx]

## Slide 1
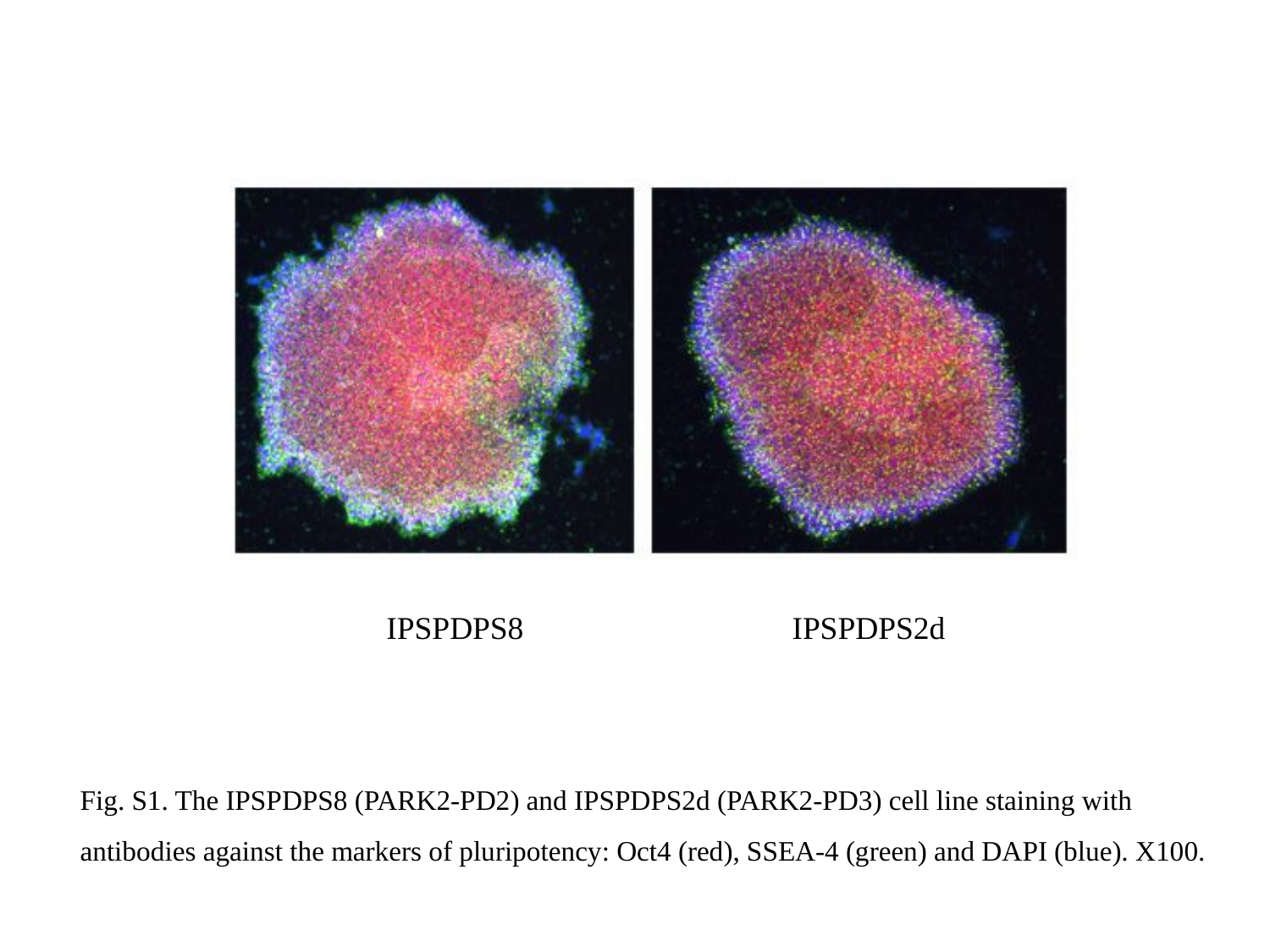

IPSPDPS8
IPSPDPS2d
Fig. S1. The IPSPDPS8 (PARK2-PD2) and IPSPDPS2d (PARK2-PD3) cell line staining with antibodies against the markers of pluripotency: Oct4 (red), SSEA-4 (green) and DAPI (blue). Х100.

## Slide 2
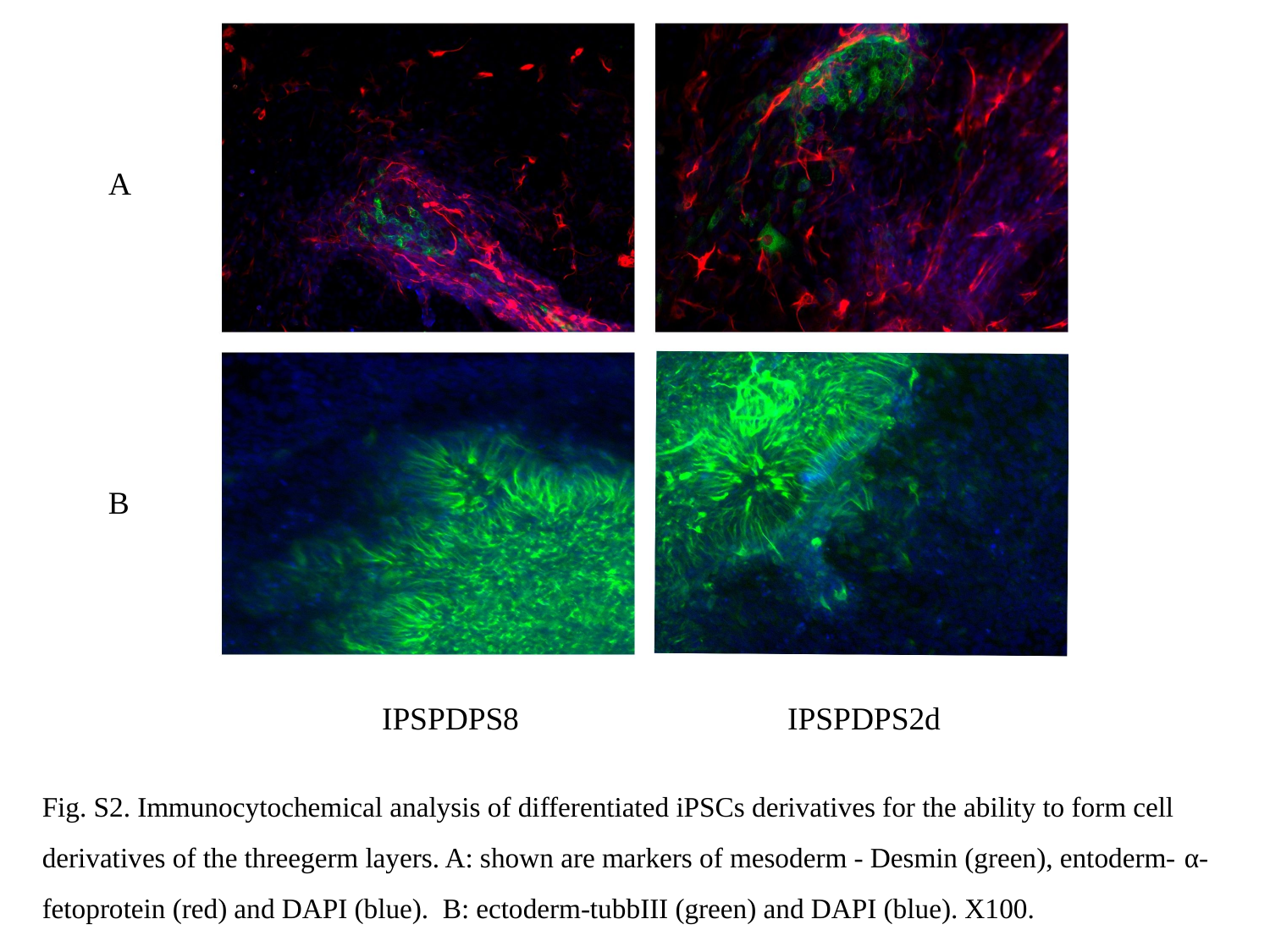

A
B
IPSPDPS8
IPSPDPS2d
Fig. S2. Immunocytochemical analysis of differentiated iPSCs derivatives for the ability to form cell derivatives of the threegerm layers. A: shown are markers of mesoderm - Desmin (green), entoderm- α-fetoprotein (red) and DAPI (blue). B: ectoderm-tubbIII (green) and DAPI (blue). X100.

## Slide 3
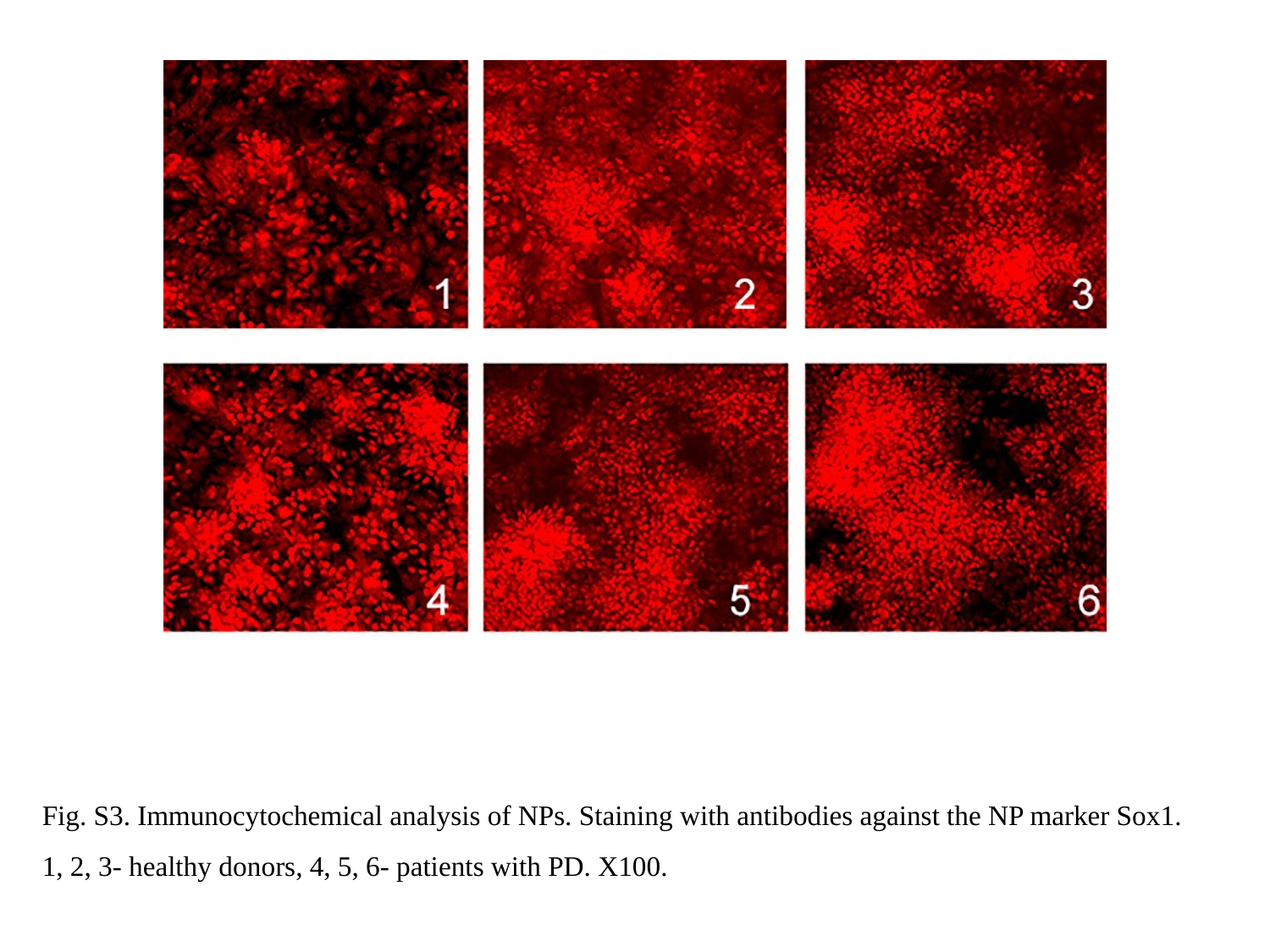

Fig. S3. Immunocytochemical analysis of NPs. Staining with antibodies against the NP marker Sox1.
1, 2, 3- healthy donors, 4, 5, 6- patients with PD. X100.

## Slide 4
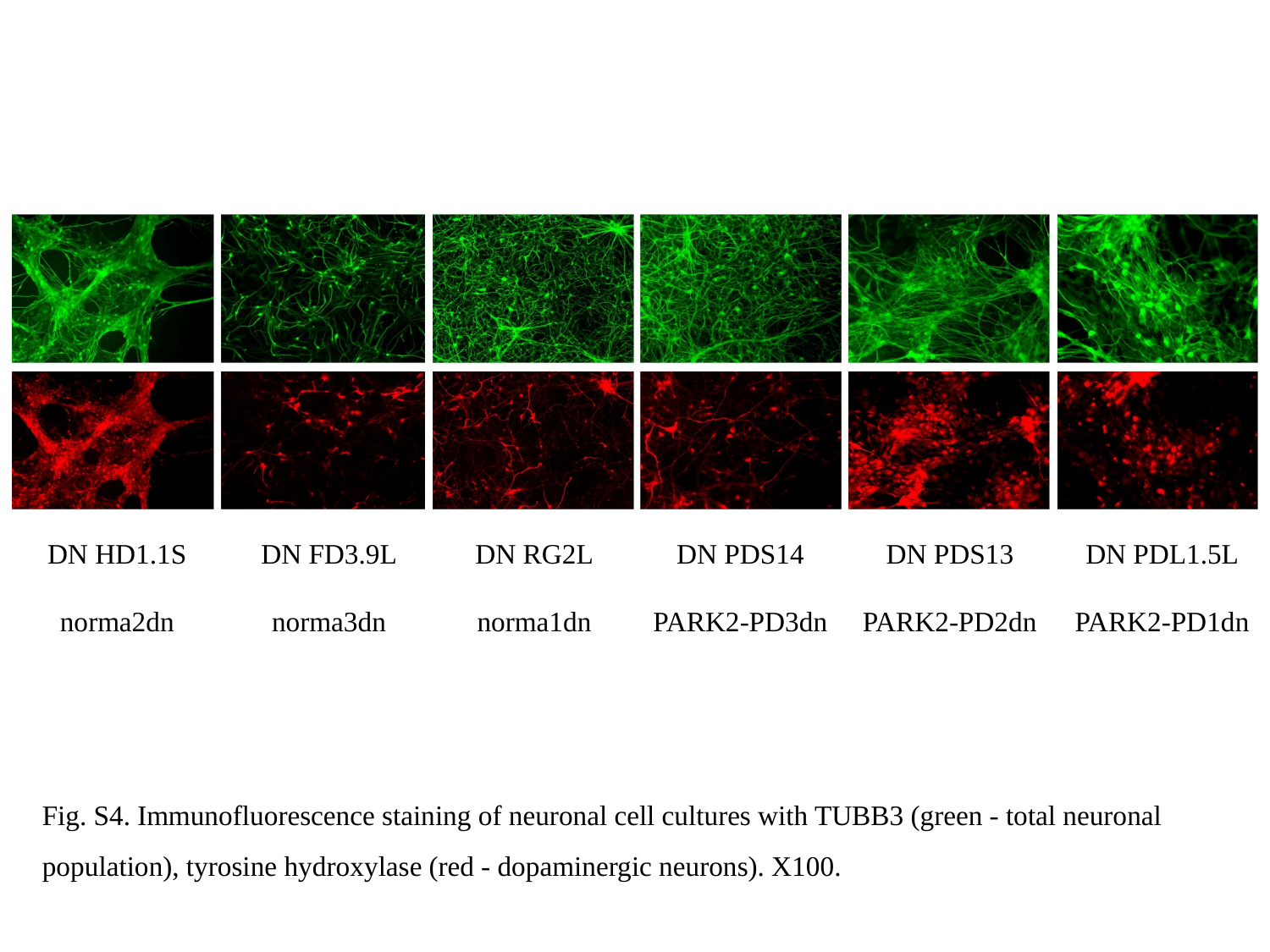

DN HD1.1S
norma2dn
DN FD3.9L
norma3dn
DN RG2L
norma1dn
DN PDS14
PARK2-PD3dn
DN PDS13
PARK2-PD2dn
DN PDL1.5L
PARK2-PD1dn
Fig. S4. Immunofluorescence staining of neuronal cell cultures with TUBB3 (green - total neuronal population), tyrosine hydroxylase (red - dopaminergic neurons). X100.

## Slide 5
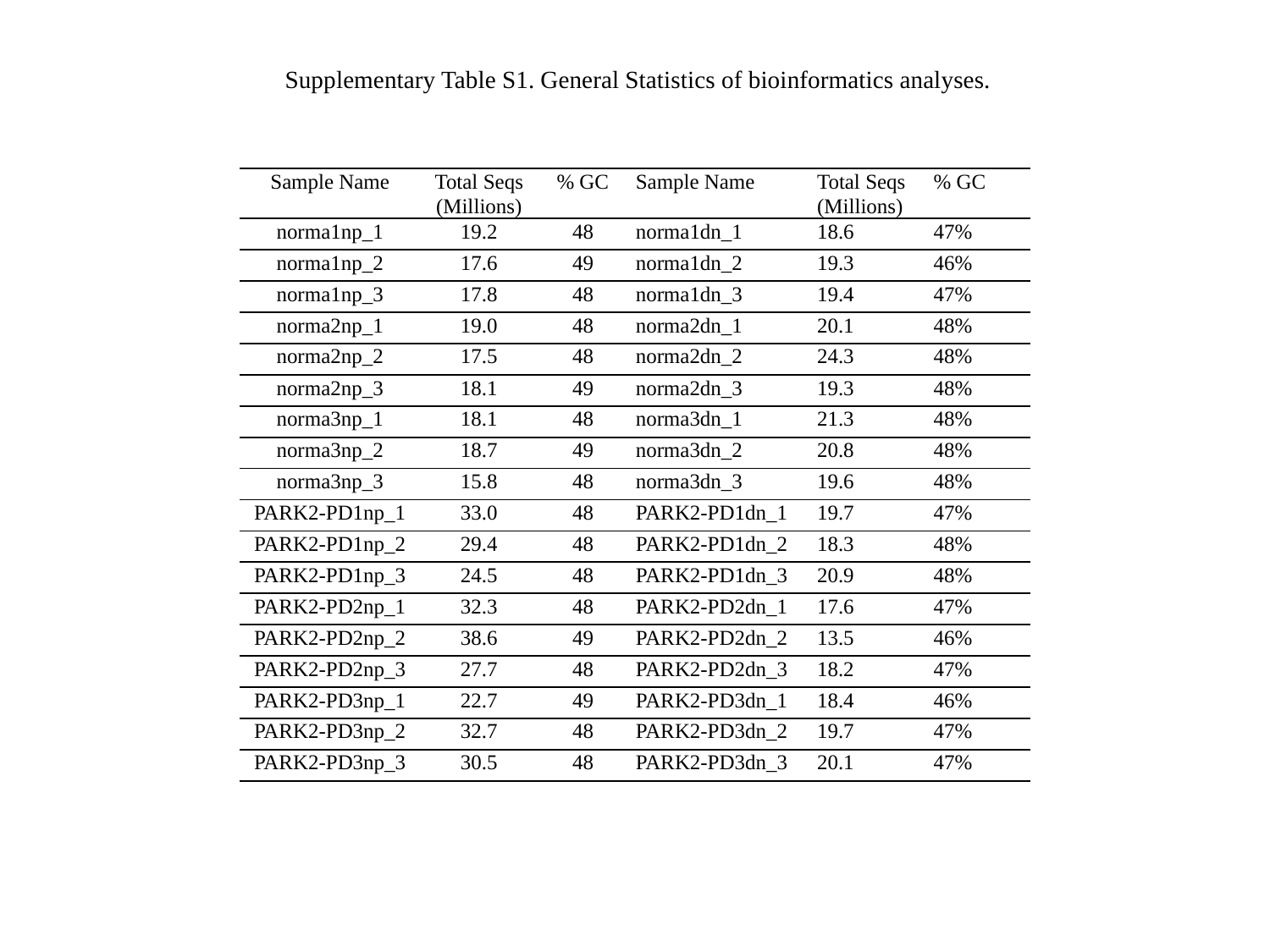

Supplementary Table S1. General Statistics of bioinformatics analyses.
| Sample Name | Total Seqs (Millions) | % GC | Sample Name | Total Seqs (Millions) | % GC |
| --- | --- | --- | --- | --- | --- |
| norma1np\_1 | 19.2 | 48 | norma1dn\_1 | 18.6 | 47% |
| norma1np\_2 | 17.6 | 49 | norma1dn\_2 | 19.3 | 46% |
| norma1np\_3 | 17.8 | 48 | norma1dn\_3 | 19.4 | 47% |
| norma2np\_1 | 19.0 | 48 | norma2dn\_1 | 20.1 | 48% |
| norma2np\_2 | 17.5 | 48 | norma2dn\_2 | 24.3 | 48% |
| norma2np\_3 | 18.1 | 49 | norma2dn\_3 | 19.3 | 48% |
| norma3np\_1 | 18.1 | 48 | norma3dn\_1 | 21.3 | 48% |
| norma3np\_2 | 18.7 | 49 | norma3dn\_2 | 20.8 | 48% |
| norma3np\_3 | 15.8 | 48 | norma3dn\_3 | 19.6 | 48% |
| PARK2-PD1np\_1 | 33.0 | 48 | PARK2-PD1dn\_1 | 19.7 | 47% |
| PARK2-PD1np\_2 | 29.4 | 48 | PARK2-PD1dn\_2 | 18.3 | 48% |
| PARK2-PD1np\_3 | 24.5 | 48 | PARK2-PD1dn\_3 | 20.9 | 48% |
| PARK2-PD2np\_1 | 32.3 | 48 | PARK2-PD2dn\_1 | 17.6 | 47% |
| PARK2-PD2np\_2 | 38.6 | 49 | PARK2-PD2dn\_2 | 13.5 | 46% |
| PARK2-PD2np\_3 | 27.7 | 48 | PARK2-PD2dn\_3 | 18.2 | 47% |
| PARK2-PD3np\_1 | 22.7 | 49 | PARK2-PD3dn\_1 | 18.4 | 46% |
| PARK2-PD3np\_2 | 32.7 | 48 | PARK2-PD3dn\_2 | 19.7 | 47% |
| PARK2-PD3np\_3 | 30.5 | 48 | PARK2-PD3dn\_3 | 20.1 | 47% |
